# NIECE AND NEPHEW DEMENTIA CAREGIVERS: FAMILY RELATIONSHIPS AND CARE DYNAMICS

**DOI:** 10.1093/geroni/igae098.1317

**Published:** 2024-12-31

**Authors:** Karen Roberto, Jyoti Savla

**Affiliations:** Virginia Tech, Blacksburg, Virginia, United States; Virginia Tech, Blacksburg, Virginia, United States

## Abstract

Changing demographics and a host of social trends have created variations in dementia family care, including older adults’ reliance on extended kin. To advance understanding of the heterogeneity of dementia family care structures, we focused on nieces and nephews who serve as primary caregivers for their aunts and uncles living with dementia. Guided by a life course perspective and grounded in a stress-coping framework, we used a mixed-method study design to explore how past and present family relationships and dynamics shape caregiving responsibilities, practices, and outcomes. Mixed-method interviews with 20 niece and 5 nephew dementia caregivers (Mage = 55 yrs; R = 38-67) revealed diverse pathways to care provision. Close relationships were established in childhood for 21 respondents, even among those who lived at a distance from their aunt/uncle. Two-thirds of the nieces/nephews never expected to be their relative’s caregiver, but rather either gradually “fell into it” or abruptly assumed the role because of “family circumstances.” Rarely were nieces/nephews the sole caregiver; some coordinated care with their aunts’/uncles’ family members, their own family members, and formal service providers whereas others looked to another family member only for occasional assistance. Nieces/nephews who relied on family support to care for their relative living with dementia experienced more caregiver burden (rs = 0.49, p<0.03) and faced greater family strain (rs = 0.61, p<0.01). Notably, niece caregivers reported more difficulties with other family members than nephews (rpb =0.50, p<0.02). Discussion elaborates on the complexity of extended family relationships and realities of dementia care today.

